# Restoring the Angiogenic Capacity of the Human Diabetic Adipose-derived mesenchymal stem cells Primed with Deferoxamine as a Hypoxia Mimetic Agent: Role of HIF-1α

**DOI:** 10.34172/apb.2023.021

**Published:** 2022-01-10

**Authors:** Raziye Tajali, Akram Eidi, Hosein Ahmadi Tafti, Abdolreza Pazouki, Ali Mohammad Sharifi

**Affiliations:** ^1^Department of Biology, Science and Research Branch, Islamic Azad University, Tehran, Iran.; ^2^Research Center for Advanced Technologies in Cardiovascular Medicine, Tehran Heart Center Hospital, Tehran University of Medical Sciences, Tehran, Iran.; ^3^Minimally Invasive Surgery Research Center, Iran University of Medical Sciences Tehran, Iran.; ^4^Stem Cell and Regenerative Medicine Research Center, Iran University of Medical Sciences, Tehran, Iran.; ^5^Razi Drug Research Center, and Department of Pharmacology, School of Medicine, Iran University of Medical Sciences, Tehran, Iran.; ^6^Tissue Engineering Group, (NOCERAL), Department of Orthopedics Surgery, Faculty of Medicine, University of Malaya, Kuala Lumpur, Malaysia.

**Keywords:** Adipose-derived mesenchymal stem cells, Angiogenesis, Deferoxamine, Type 2 diabetes

## Abstract

***Purpose:*** Insufficient angiogenesis is associated with serious diabetic complications. Recently, adipose-derived mesenchymal stem cells (ADScs) are known to be a promising tool causing therapeutic neovascularization. However, the overall therapeutic efficacy of these cells is impaired by diabetes. This study aims to investigate whether in vitro pharmacological priming with deferoxamine, a hypoxia mimetic agent, could restore the angiogenic potential of diabetic human ADSCs.

***Methods:*** Diabetic human ADSCs were treated with deferoxamine and compared to normal and nontreated diabetic ADSCs for the expression of hypoxia inducible factor 1-alpha (HIF-1α), vascular endothelial growth factor (VEGF), fibroblast growth factor-2 (FGF-2) and stromal cell-derived factor-1α (SDF-1α), at mRNA and protein levels, using qRT-PCR, western blotting and ELISA assay. Activities of matrix metalloproteinases (MMPs)-2 and -9 were measured using a gelatin zymography assay. Angiogenic potentials of conditioned media derived from normal, Deferoxamine treated, and non-treated ADSCs were determined by in vitro scratch assay and also three-dimensional tube formation assay.

***Results:*** It is demonstrated that deferoxamine (150 and 300 μM) stabilized HIF-1α in primed diabetic ADSCs. At the concentrations used, deferoxamine did not show any cytotoxic effects. In deferoxamine treated ADSCs, expression of VEGF, SDF-1α, FGF-2 and the activity of MMP-2 and MMP-9 were significantly increased compared to nontreated ADSCs. Moreover, deferoxamine increased the paracrine effects of diabetic ADSCs in promoting endothelial cell migration and tube formation.

***Conclusion:*** Deferoxamine might be an effective drug for pharmacological priming of diabetic ADSCs to enhance the expression of proangiogenic factors noted via HIF-1α accumulation. In addition, impaired angiogenic potential of conditioned medium derived from diabetic ADSCs was restored by deferoxamine.

## Introduction

 Much of the deaths and morbidity related to type 2 diabetes mellitus (T2DM) predominantly reflects its negative effects on the physiological angiogenesis.^1^ Insufficient angiogenesis acts in the peripheral vasculature and involves in the impaired wound healing, cardiac mortality, as well as worsened peripheral limb ischemia.^2^ In this regard, there is a rationale for targeting angiogenesis to restore normal circulation in T2DM.

 Autologous cell therapy with mesenchymal stem cell (MSC), particularly adipose-derived mesenchymal stem cells (ADSCs), is an attractive therapeutic intervention for treating diabetic complications, due to the ease and efficiency of acquisition, their ability for differentiation into a variety of distinct cell lineages, secretion of anti-inflammatory, anti-scarring and anti-apoptotic molecules, and growth factors that support angiogenesis, immunomodulation and protection from apoptotic cell death.^3^ However, some studies indicated that the proliferation, survival, and angiogenic capacity of ADSCs derived from diabetic patients may be impaired compared to their nondiabetic counterparts.^4^ Therefore, strategies for reviving intrinsic impaired mechanisms before cell implantation have become one of the hot topics of central attention.

 According to a study in the field, hypoxia-inducible factor 1 (HIF-1) has been proposed as one of the key mediator regulating genes, which contributes to several processes promoting neovascularization and makes regulation of the HIF expression one of the promising approaches for therapeutic revascularization.^5^ It consists of two subunits, a hypoxia-regulated alpha sub-unit as well as one of the stable beta subunits. It is well established that under normoxic condition, HIF-1α is rapidly hydroxylated in the oxygen-dependent degradation domain through certain prolyl hydroxylases (PHDs),^6^ which is accompanied by proteasomal degradation and ubiquitination.^7^ In fact, PHDs are a group of enzymes that need oxygen and 2-oxoglutarate as the co-substrates, and ascorbate and iron (Fe2 + ), as co-factors.^8^ In normoxia, using an iron chelator causes PHD inhibition, allowing the stabilization of HIF-α, and its binding to hypoxia-responsive elements (HREs) in regulatory areas of the target genes like vascular endothelial growth factor (VEGF), stromal cell-derived factor-1α (SDF-1α), fibroblast growth factor-2 (FGF-2) and matrix metalloproteinases (MMPs)-2 and -9.

 Many researchers have attempted to modulate the paracrine actions of ADSCs with different pharmacological molecules including melatonin,^9^ vitamin E,^10^ vitamin C,^11^ rosuvastatin,^12^ docosahexaenoic acid,^13^ dimethyloxalylglycine,^14^ curcumin,^15^ and Exendin-4^16^ to enhance their therapeutic efficacy. Deferoxamine (DFO) is a potent prolyl-hydroxylase inhibitor (PHDi) which inhibits the degradation of HIF-1α at a normoxic condition.^17^ Consecutively, this favors augmentation of HIF-1α, which leads to nuclear translocation, dimerization with HIF-1β and transactivation of several critical angiogenic factors, like SDF-1α, FGF-2 and VEGF.^18^ Utilizing this mechanism, DFO also enhances angiogenesis with a concentration and time-dependent method in ADCSs.^19^ Furthermore, deferoxamine exhibited a similar effect on diabetic ADSCs to restore angiogenic potential in animal models.^20^

 This study sought for the evaluation of invitro pre-conditioning of human diabetic ADSCs with DFO was able to restore the angiogenic potential of these cells or not as there are currently no reports exploring this. We believe that our findings may be useful in the development of autologous cell therapy in T2DM.

## Materials and Methods

###  Antibodies and reagents

 Minimal essential medium α (αMEM), penicillin–streptomycin and fetal bovine serum (FBS) were from Gibco (Invitrogen, Carlsbad, CA). All materials for differentiation assay, phosphate-buffered saline (PBS), 3-(4, 5-dimethylthiazol-2-yl)-2, 5-diphenyltetrazolium bromide (MTT), NaCl, KCl, HEPES, EDTA, Collagenase type I, Deferoxamine, gelatin A and B were provided from Sigma (Sigma Aldrich; St Louis: MO). StemPro® Chondrogenesis Differentiation Kit was from Thermo Scientific (Wilmington, DE, USA). Dimethyl sulfoxide (DMSO) was purchased from (Carl Roth GmbH & Co., Karlsruhe: Germany). Fluorescein isothiocyanate (FITC)-conjugated mouse anti-human against CD105, CD90, CD73, CD31, CD34, and HLA-DR were purchased from eBioscience (USA). Trizol reagent was from Invitrogen (Merelbeke, Belgium). The antibodies were from the Abcam (UAS). QuantiNova SYBR Green PCR Master Mix was purchased from QIAGEN (Germany) and qPCRBIO cDNA synthesis kit was obtained from PCR Biosystems (London, UK). RIPA Lysis buffer and phenyl methane sulfonyl fluoride (PMSF) were obtained from Roche (Applied Science, Penzberg, Germany). Polyvinylidene fluoride (PVDF) membrane was purchased from BioRad (Hercules, CA). Chemiluminescence (ECL) kit was from Amersham Biosciences (Buckinghamshire, UK). Horseradish peroxidase‐linked anti‐rabbit secondary antibody and anti‐glyceraldehyde‐3‐phosphate dehydrogenase antibody (GAPDH) were obtained from Cell Signaling (Danvers, MA, USA).

###  Isolation, culturing, and characterization of the human ADSCs

 According to the research design, we isolated human ADSCs from the samples of sub-cutaneous adipose tissue provided by 6 adult patients with T2DM and 3 healthy adults at the Atieh Hospital, Tehran, Iran, after obtaining written informed consents (Table 1). Each experimental protocol was confirmed by the ethical committee of Iran University of Medical Sciences.

**Table 1:** Clinical characteristics of ADSCs donors

**Clinical characteristic**	**Groups of patients**		***P***
	**Normal **	**Diabetic**	
	**n=3 **	**n=6**	
Gender (female/male)	2/1	4/2	
Age, year	37 ± 5.8	59 ± 6.94	0.0412
FBS	113.33 ± 8.49	181 ± 46.07	0.169
HbA1c	5.16 ± 0.49	9.06 ± 1.40	0.046
BMI	42.33 ± 2.49	37.33 ± 2.62	0.122

Data are shown as mean ± SD. *P* value for comparing healthy and diabetic donors using Mann-Whitney U test.

 For ADSC isolation, we digested samples of the sub-cutaneous adipose tissue (0.5-5 mL) with 150 μg/mL (0.075%) collagenase type I prepared in the αMEM through stirring at 37 °C for 30-40 minutes. Then, centrifuge of the mixture was done at 470 × g for ten minutes and we lysed the pellet consisting of ADSC for destroying erythrocytes, sieved via a 100 mm cell strainer, and finally centrifuged it at 470 × g for ten minutes. Afterwards, we re-suspended the resulting pellet and cultured it in αMEM, with the supplements: 1% penicillin/streptomycin and 10% FBS based on the acceptable cell-culture conditions; that is, 37°C and 5% CO_2_.^21^ Each experiment was performed with the cells from passage three 3 times (n = 3). In order for evaluating the adipogenic differentiation potential, we incubated the cells’ culture (passage 3) using 10−9 M of dexamethasone (C_22_H_29_FO_5_) and 5 μg/mL insulin. After 14 days of stimulation, Oil Red O staining verified adipogenic differentiation for visualization of lipid droplets. For osteogenesis assay, third passage cells with the osteogenesis medium (10 mM β-glycerophosphate, 109 M C_22_H_29_FO_5_, 50 μg/mL ascorbic acid 2-phosphates) for 14 days. Mineralization of ECM was evaluated via staining with Alizarin Red. For assessment of chondrogenic potential, incubation of the cells was performed with 5% CO2 in the Dulbecco’s modified Eagles medium (DMEM)-high glucose and chondrogenesis differentiation medium with chondrogenesis supplementation at 37°C. After 21 days, we used Alcian blue staining for assessing chondrogenic differentiation and applied caliber cytometer FACS (Becton Dickinson; San Diego; CA: USA) for flowcytometry for analyzing the third-passage MSCs surface markers like CD31, CD34, HLA-DR, CD105, CD90 and CD73.

###  Collection and concentration of ADSC conditioned medium (CM)

 ADSCs at 80–90% confluence in 6-well plates were washed three times with PBS and were cultured with 3 mL fresh serum-free α-MEM for 24 hours. then conditioned medium was collected and concentrated 50-fold with the use of the ultrafiltration centrifugal filter-units through a 3 kDa cutoff (Millipore; Bedford, MA, USA) as illustrated in the company’s directions.^22^

###  Cytotoxicity assay

 In this step, we plated the cells (5 × 10^3^) cells in 96-well microplate and treated them using a medium consisting of with 0, 75, 150 and 300 μM of DFO. When incubation of the cells was performed for 24 and 48 hours, we poured 100 μL MTT 3- (4, 5-dimethylthiazol-2-yl)-2, 5-diphenyltetrazolium bromide) reagent to each well. Following incubation in the incubator with a temperature of 37°C under 5% CO2 for one hour, we poured 100 μL dimethyl sulfoxide (DMSO) into the wells. Finally, we used a micro-plate reader (Bio-Tek ELX800; Winooski, VT, USA) to measure the media absorption at 570 nm.^20^

###  qRT -PCR

 According to the research design, we extracted the total RNA from normal, diabetic and pretreated diabetic ADSCs when they have been exposed to DFO with the use of a TRIzol reagent based on the company’s protocol.In the next step, we employed a nanodrop 2000 spectrophotometer (Thermo Scientific; Wilmington, DE, USA) to evaluate RNA’s quality and concentration. Moreover, we used reverse transcriptase with a cDNA synthesis kit to synthesize cDNA. Samples were analyzed by a Rotor-Gene Q 5plex HRM System and the reaction mixture consisted of 12 μL of QuantiNova SYBR Green PCR Master Mix, a cDNA template, as well as particular primers like: reverse (5′-TGCATTCACATTTGTTGTGCTGTAG-3′), SDF-1α; VEGF; forward (5′-TGCAGATTATGCGGATCAAACC-3′) and forward (5′-CCCACAAATCACAGGCATAG-3′) and reverse (5′-GTGCCCTTCAGATTGTAGCC-3′); FGF-2; forward (5′-AGCGGCTGTACTGCAAAAACGG-3′) and reverse (5′-CCTTTGATAGACACAACTCCTCTC-3′) and β-actin (as a loading control); forward ((5′-TGTCCACCTTCCAGCAGATGT-3′) and reverse (5′-AGCTCAGTAACAGTCCGCCTAGA-3′). Finally, we defined the threshold cycle (Ct) for target genes of all samples and β-actin and computed the relative expression of the genes for all samples.^23^

###  Western blot analysis

 To perform total protein extraction, we trypsinized the normal, diabetic and treated ADSCs and washed them two times in cold PBS. Then, we lysed the cells with 1 × RIPA lysis buffer consisting of a protease and phosphatase inhibitor cocktail.^24^ Moreover, we discarded the cell debris via centrifuging at 12000 × g with a temperature of 4°C for thirty minutes, collected the supernatant, and kept at a temperature of −80°C. In addition, we employed Bradford assay (Bio-Rad Laboratories; Hercules, CA, USA) for determining the protein concentration.^25^ Equal concentration of the samples (150 µg) was isolated by SDS-PAGE on a 10% gel and transported to a polyvinylidene difluoride (PVDF) membrane. Furthermore, certain antibodies like anti-HIF- 1α (1: 1000), anti-β Actin (1: 2000) antibody and anti-SDF- 1 (1: 1000) as one of the internal controls were utilized to incubate the membrane. Incubation of the membrane was performed with a secondary antibody, which has been conjugated to horseradish peroxidase, at the room temperature for 1 hour. Then, we used an enhanced chemiluminescence kit for visualizing the protein bands and subsequently quantified with an ImageJ software (National Institutes of Health, Bethesda, MD, USA).^24^

###  Quantification of protein levels of VEGF and FGF-2

 VEGF and FGF-2 concentrations were quantified with a human Quantikine ELISA kit (R&D Systems) based on the company’s directions. Then, we used a microplate reader (Molecular Devices, Sunnyvale, CA, USA) to measure absorbance at 450 nm as well as a standard curve to calculate the concentration.

###  Gelatin zymography 

 To evaluate the effect of DFO on the matrix metallo-proteinases (MMPs)-2 as well as -9 activities, we procured normal ADSCs and diabetic ADSCs CM as aforementioned and exposed them to zymographic assay. After that, we blended the supernatants with the nonreducing sample buffer (0.5 M Tris/HCl, 10 % (w/v), pH 6.8, SDS, 10 % (v/v) glycerol and 0.02 % (w/v) bromophenol blue). Thereafter, we separated the mixtures electrophoretically with 10% poly-acrylamide gel in the presence of 0.1% SDS consisting of B (MMP-9) or 0.1% gelatin A (MMP-2). Following the electrophoresis process, we washed gels two times in 2.5% Triton X-100 at room temperature for thirty minutes, to remove SDS and performed incubation overnight in a developing buffer (50-mM Tris-HCl, pH 8, 2-mM CaCl2, 0.02% NaN3) at 37°C. The following day, Coomassie Blue solution containing 40% methanol, 0.1% Coomassie Brilliant Blue G-250, 10% acetic acid and deionized water was used to stain the gels for 2 hours at room temperature and subsequently de-stained in the same solution without any dye. A transparent zone in the blue background reflected the existence of gelatinolytic activities. Then, we compared the protein molecular weight standards (Bio-Rad) for determining the molecular weights of proteinases and ultimately used NIH ImageJ software to photograph and analyze the gels.^20^

###  In vitro scratch assay

 For examining the wound healing impact of the normal, diabetic, and DFO treated CMs on the human umbilical vein endothelial cells (HUVECs), as considered in the research design, we seeded the HUVECs into a six-well plate at 5 × 10^5^ cells/well. Upon a 24-hour incubation, all of the confluent monolayers were scratched with a sterile 1000 μL plastic pipette tip for creating a wounded cell-free area. After that, we washed them two times with PBS. Then, 2 mL of CM extracted from the treated and nontreated ADSCs were added, followed by incubation at 37°C. Then, time-lapse photography was used to monitor wound healing, which took images per hour in the course of the cell migration from 5 random, individual microscopic fields and analyzed them with a NIH ImageJ software.^26^

###  HUVEC capillary tube formation in 3-dimensional (3D) collagen gel

 In this step, we blended the HUVECs with Cytodex-3 micro-carrier beads and put them in the incubator at a temperature of 37°C. Then, we flicked the mixed suspension every 20 minutes for uniformly distributing the cells over the micro-carrier beads. Afterwards, we seeded the beads in the 12-well plates and incubation was done at a temperature of 37°C overnight. For the next day, we put the cell-coated beads in the serum-free medium and ice-cold collagen matrix and loaded the mixture into a 96-well plate in order to be solidified at 37°C for 45 minutes. Afterwards, we poured the serum-free medium as well as the CMs extracted from the normal, DFO treated, and nontreated diabetic ADSCs into the wells and photographed the cells after 48 hours. Finally, NIH ImageJ software was used to analyze the sprout formation based on the standard method and expressed as the percentage of control.^27^

###  Statistical analyses

 Data were shown as the mean ± standard deviation (SD). Moreover, we employed one-way analysis of variance (ANOVA) for comparing the mean values among several treatment groups with a Tukey post hoc multiple comparison test. In addition, we considered statistical significance at *P* < 0.05.

## Results and Discussion

###  Characterization and morphology of human ADSCs

**Figure 1:**
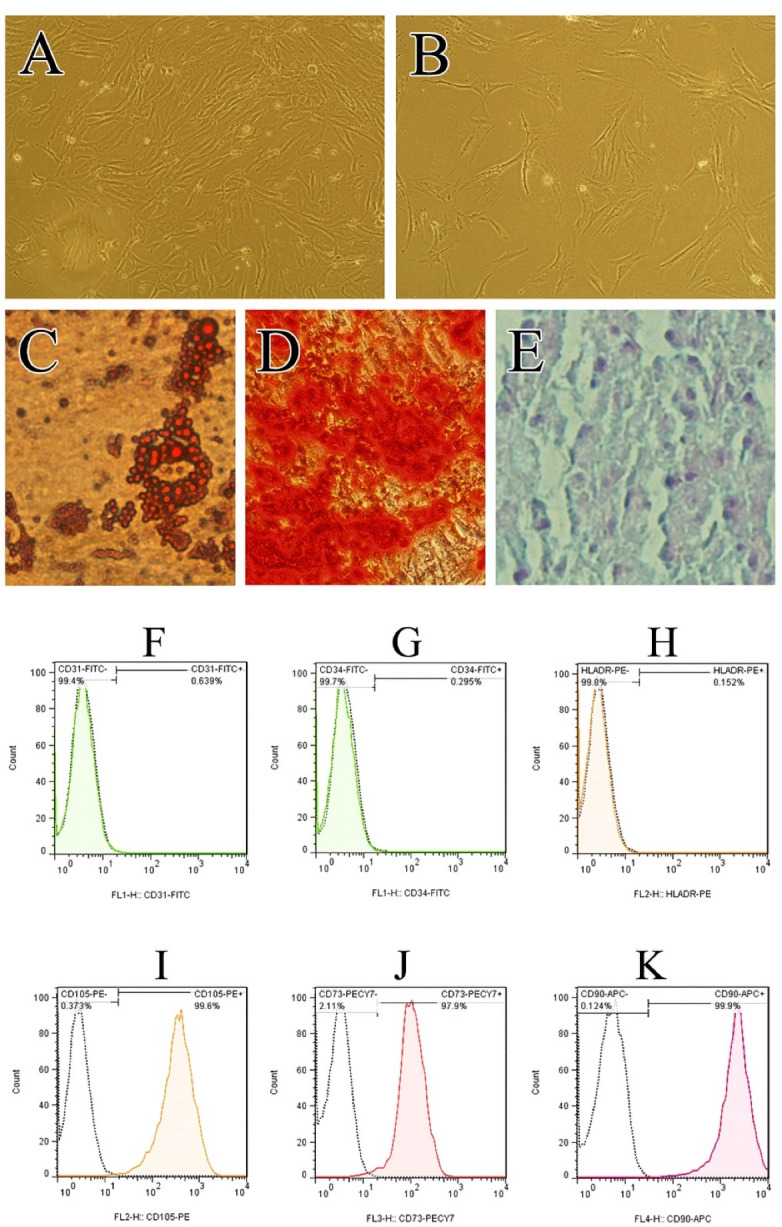
Characterization of hADSCs. Photomicrographs show the morphology of cultured ADSCs at passage 3 prepared from Normal (A) and Diabetic donors (B). Light microscopy images of hADSCs differentiation into the adipocytes (Oil Red staining) (C), osteoblasts (Alizarin Red staining) (D), and chondrocytes (Alcian blue staining/nuclear fast red counterstain) (E). Flowcytometry analysis of hADSCs at passage 3 for putative human MSC markers (CD105, CD73, CD90 & CD105) as well as markers which are the characteristic of other cell-lineages (CD31, CD34 & HLA-DR) (F-K).

 ADSCs were successfully isolated from all three healthy specimens collected. However, isolation of ADSCs was successful for 3 out of 6 samples (50%) obtained from diabetic patients. Normal ADSCs exhibited a spindle-shaped morphology (Figure 1A). On the other hand, diabetic ADSCs became enlarged and showed flattened morphology (Figure 1B). This change in the diabetic ADSCs morphology was similar to the reported effect of diabetes on rat ADSCs.^20^ Furthermore, enlarged individual cell area and flat shape with increased actin filaments in the cytoplasm were observed in rat diabetic BMSCs.^28,29^ It has been demonstrated that diabetes powerfully affects MSC morphology, including actin cytoskeleton organization.^30^ It is believed that this morphological change is highly associated with increased apoptosis, as well as senescence.^31,32^ This is probably due to excessive accumulation of oxidative stress factors and reduced antioxidative defense in diabetic MSCs.

 ADSCs showed the ability for differentiating into osteoblasts, chondrocytes as well as adipocytes (Figure 1C-E), when they were cultured in appropriate culture conditions. ADSCs at passage three were characterized based on the International Society for Cellular Therapy criteria to determine the multi-potent mesenchymal stromal cells.^33^ Flow cytometry analysis demonstrated that ADSCs were positive for CD105, CD73, and CD90 (Figure 1I-K) but negative for the hematopoietic markers CD31, CD34 and HLA-DR (Figure 1F-H).

###  Concentration responses and time course of the DFO treatment on viability of human ADSCs

**Figure 2:**
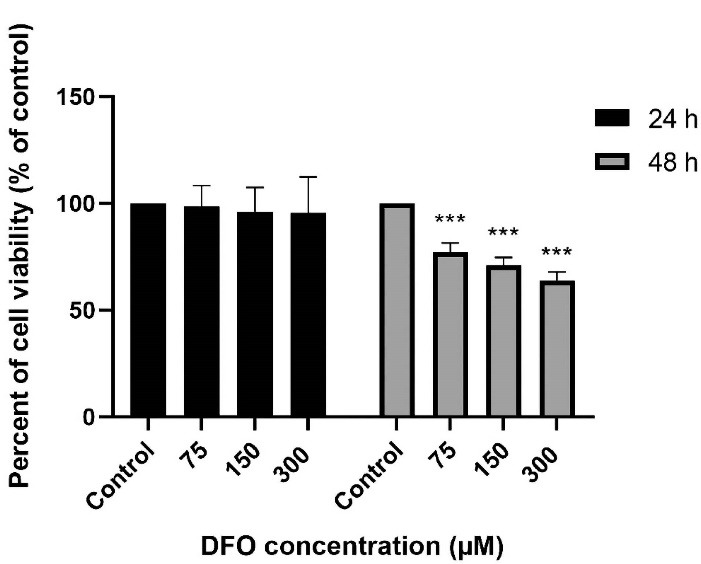
DFO effects on the ADSCs' cell viability. Diabetic ADSCs were treated with 75, 150 as well as 300 µM of DFO for 24 hours and 48 hours. Then, MTT colorimetric assay was used to measure viability. As seen, data have been written as the mean ± SEM (n = 5; ***P < 0.001 versus non treated diabetic cells) and repeated measures ANOVA was used to measure statistical significance.

 For finding the optimum time for early treatment with DFO as well as an optimal concentration, we incubated the diabetic ADSCs with 75, 150, and 300 µM of DFO. Analysis showed that a 24-hour pre-conditioning with DFO would not lead to the induction of the changes in the cells’ morphology and thus would not show significant cytotoxicity. Put differently, DFO resulted in 22.9%, 28.9% and 46.2% cytotoxicity at a concentration of 75, 150 and 300 µM after 48 hours (Figure 2). Therefore, we used DFO at concentrations equal to 150 and 300 µM for 24 hour in all other tests of the research.

###  Deferoxamine pre-conditioning enhanced VEGF, SDF-1α and FGF-2 mRNA level of expression

**Figure 3:**
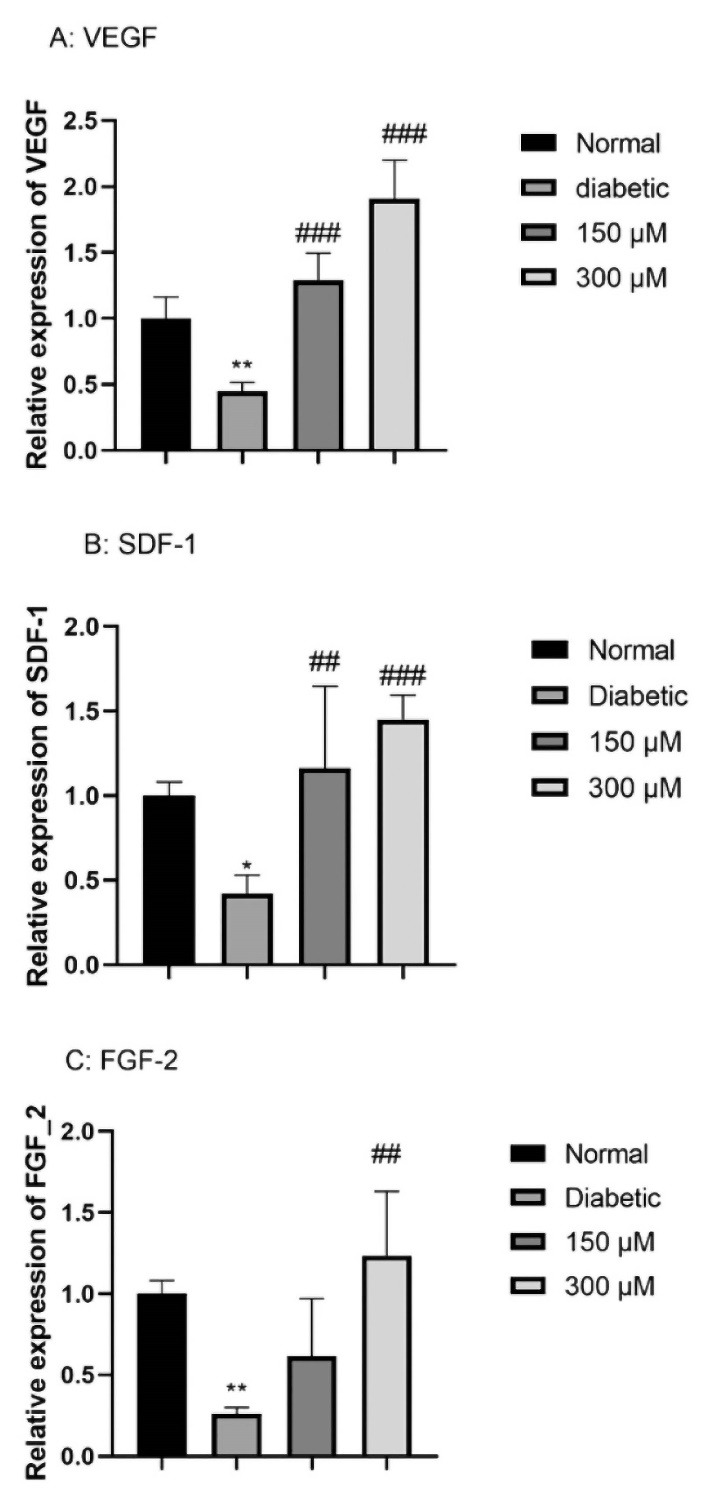
The effects of DFO (150 and 300 μM) on the level of expression of the angiogenic gene in Diabetic ADSCs. Then, we preconditioned the cells with DFO (150 and 300 μM) for 24 h and defined the level of expression of gene by qRT-PCR: (A) VEGF; (B) SDF-1α, and (C) FGF-2. As mentioned earlier, data are expressed as the mean ± SEM (n = 3; *P < 0.05, **P < 0.01 versus the normal cells, and ##*P* < 0.01, ###*P* < 0.001 versus the diabetic cells. Finally, we applied one-way ANOVA to measure statistical significance.

 In this step, we run the quantitative real-time PCR on the normal, DFO-treated and non-treated ADSCs. According to the findings, for each gene, diabetic ADSCs significantly declined the respective mRNA levels in comparison with the normal ADSCs; hence, VEGF, SDF-1, and FGF-2 level of expression showed 0.25 ± 0.002 (*P* < 0.01), 0.44 ± 0.004 (*P* < 0.01), and 0.42 ± 0.006 (*P* < 0.05) times lower in diabetic ADSCs. Moreover, pre-conditioning of diabetic ADSCs with 150 μM DFO or 300 μM DFO considerably enhanced level of mRNA of the proangiogenic factors VEGF, SDF-1α and FGF-2. (for VEGF: 1.16 ± 0.01 folds of change for 150 μM DFO pre-conditioned cells and 1.90 ± 0.01 folds of change for 300 μM DFO pre-conditioned cells; for SDF-1α: 1.16 ± 0.03 folds of change for 150 μM DFO pre-conditioned cells and 1.45 ± 0.01 folds of change for 300 μM DFO pre-conditioned cells; for FGF-2:.61 ± 0.02 folds of change for 150 μM DFO pre-conditioned cells and 1.23 ± 0.02 folds of change for 300 μM DFO pre-conditioned cells (Figure 3).

###  Deferoxamine preconditioning enhanced protein expression of HIF-1α and proangiogenic factors

**Figure 4:**
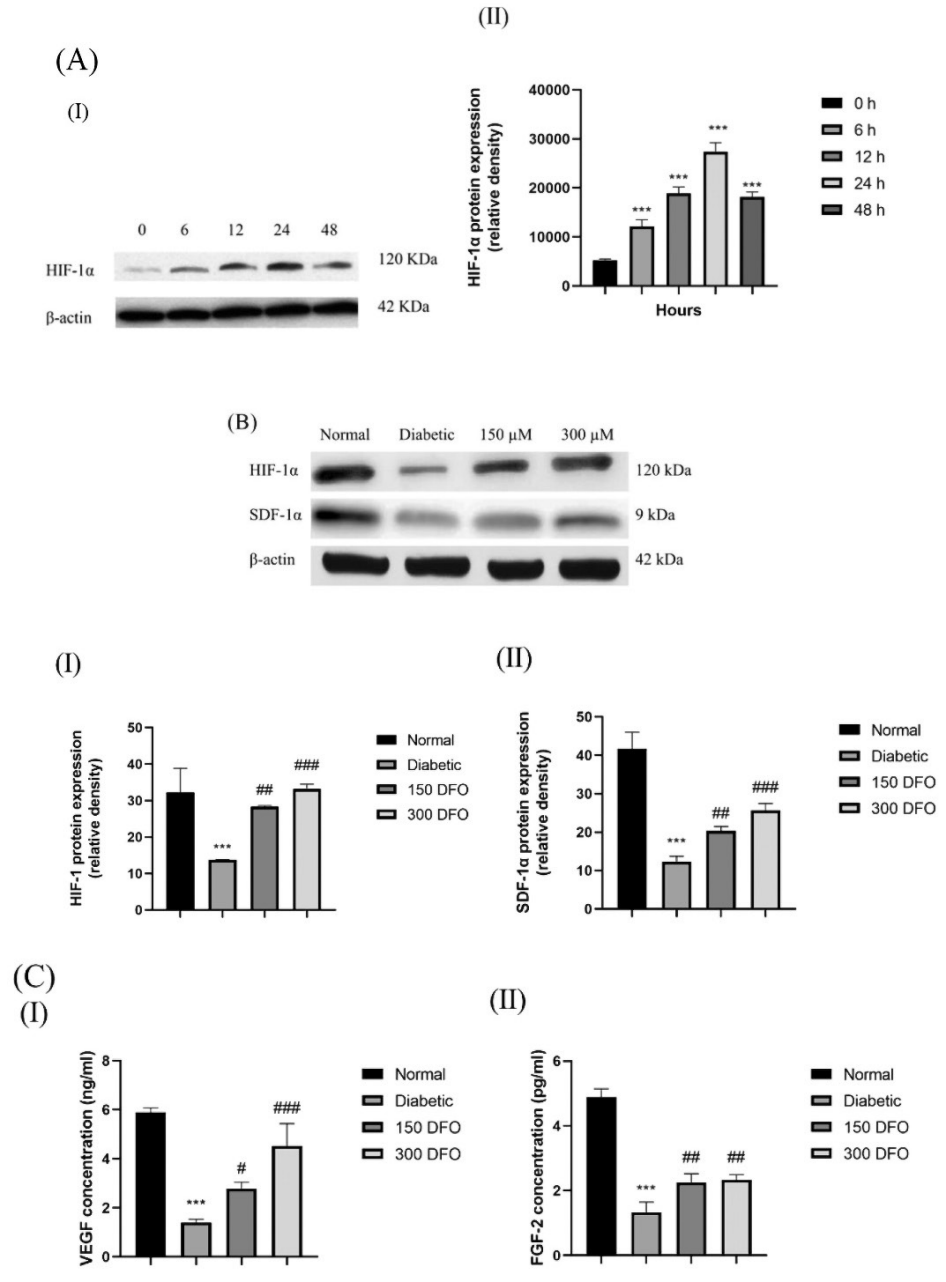
(A) Characteristic blot (I) and semi-quantitative data (II) of the level of expression of HIF-1α in the diabetic ADSCs pretreated with 150 μM DFO during a proper interval of 0, 6, 12, 24, and 48 h. (B) Characteristic blots (I) and semiquantitative data (II & III) of expression of HIF-1α and SDF-1α at the level of protein in normal, diabetic, and treated ADSCs with various concentrations of DFO following 24 h. (C) Secretion measurement of VEGF (I) and FGF-2 (II) in CM of the ADSCs when treated with DFO through ELISA assay. As mentioned earlier, data would be written as the mean ± SEM (n = 3; **P < 0.01, ***P < 0.001 versus the normal cells, and #*P* < 0.05, ##*P* < 0.01, ###*P* < 0.001 versus the diabetic cells). Finally, we employed one-way ANOVA to measure the statistical significance.

 Western blotting was carried out for evaluating HIF‐1α expression in DFO treated ADSCs with 150 μM DFO over the appropriate time period (0, 6, 12, 24 hours). DFO significantly increased HIF‐1α expression after 6 h and to a highest level after 24 hours, before showing cytotoxic effects (Figure 4A).

 Western blotting was also carried out for comparing the expression of HIF-1α and SDF-1α protein in normal ADSCs, DFO-treated and nontreated diabetic ADSCs. With regard to the findings, diabetic ADSCs showed considerably a low expression of HIF-1α and SDF-1α protein in comparison with the normal ADSCs (*P* < 0.001) (Figure 4B).

 In order to evaluate whether the increases in the mRNA levels of VEGF and FGF-2 were paralleled by increases in the corresponding, ELISA were carried out on secretomes derived from normal, diabetic and DFO preconditioned ADSCs. We observed that secretion of FGF-2 and VEGF in the CM of diabetic ADSCs remarkably declined in comparison to the one observed in the normal ADSCs. Moreover, incubating diabetic ADSCs with DFO (150 μM or 300μM) significantly enhanced the protein level of the pro-angiogenic factor VEGF and FGF-2 in comparison with the level observed in the secretomes of the non-pre-conditioned diabetic ADSCs (Figure 4C).

 The obtained results stay in good match with the earlier investigations which showed diabetic conditions decrease the secretion of angiogenic cytokines.^4,34^ Reduced VEGF, SDF-1α, and FGF-2 levels in diabetic ADSCs point to a predominantly defective abilities in the production and release of the cell mediators. Specifically, these variables contribute crucially to angiogenesis and wound healing and strongly released by the ADSCs.^35^

 The impaired mechanisms reported in the ADSCs separated from the diabetic donors can determine the lower therapeutic efficiency of the cells in cell therapy for diabetic patients. To address this issue, we preconditioned diabetic ADSCs with deferoxamine as one of the hypoxia mimetic agents for restoring angiogenesis activity of these cells. We found that DFO Preconditioning of diabetic ADSCs upregulates expression of HIF-1α at the protein level, leading to the enhanced angiogenic potency of these cells. This result is in accordance with those of previous studies that were conducted on rat diabetic ADCSs,^20,36^ mouse diabetic Bone marrow stem cells,^37^ and normal human ADSCs.^38,39^ Moreover, in the presence of oxygen and iron, it was found that HIF-1α was ubiquitinated and then targeted for proteasomal degradation via the PHD/VHL pathway. It is thought that deferoxamine, a PHDi, may stabilize HIF-1 by iron chelation.^40^ Elevated HIF-1α leads to an upregulation of the genes associated with the pro-angiogenic responses like VEGF, SDF-1α, as well as FGF-2.^41^

 However, the impact of deferoxamine on the levels of expression of these aforementioned genes in human ASCs isolated from diabetic patients have not been investigated previously. We found that DFO preconditioning accordingly increased the expression of FGF-2, SDF-1α as well as VEGF in mRNA and the protein level. These results are supported by earlier studies indicating that DFO remarkably enhanced the VEGF expression in a HIF-1α-dependent way in various cell types, such as normal human ADSCs,^39^ bone marrow stromal cells (BMSCs),^42^ and pancreatic beta cells.^43^ Similarly, Wang et al demonstrated local injection of DFO promotes wound healing in the diabetic skin flaps in mice via enhancing expression level of VEGF and HIF-1α.^44^ Additionally, it has been reported that DFO enhances neovascularization through upregulation of HIF-1α as well as VEGF and SDF-1α in an excisional diabetic wound model.^45^ In fact, SDF-1α contributes importantly to vasculogenesis that is used as the homing signals to mobilize and recruit the vascular progenitor cells from distant locations. Chekanov and Nikolaychik showed that DFO increased expressing FGF-2 in the fibroblasts and smooth muscle cells.^46^ On the contrary, Potier and colleagues’ investigation illustrated that pretreating of BMSC with deferoxamine has no significant effect on FGF2 expression.^47^ This discrepancy can be clarified in this way: previous studies used different cells. Moreover, it may be attributable to the difference in concentration and time of DFO used for preconditioning. Further study would be crucial to widely and comprehensively explore the impact of platelet micro vesicles on the secretome of endothelial cell.

###  Deferoxamine preconditioning enhanced MMP-2 and 9 activity in the ADSCs conditioned media

 Matrix metalloproteinases (MMPs) are one of the families of Zn2 + endopeptidases that engage in the degradation of extra-cellular matrix (ECM).^48^ Among these, Matrix metalloproteinase 2 and 9 (MMP-2 & MMP-9) or gelatinases are highlighted because of their significant proteolytic role during the initial phase of angiogenesis.^49^ Previous studies demonstrated that hypoxic conditions up-regulate MMP family by a HIF-1α-dependent pathway.^50^

**Figure 5:**
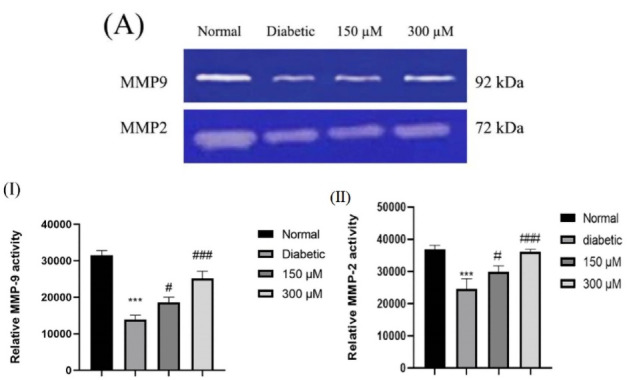
(A) Characteristic gelatin zymograms of MMP-9 and MMP-2 activities in the ADSCs. Those regions of protease activities are marked by white bands. Densitometry (I & II) was used to perform quantitative analysis. Data are written as the mean ± SEM (***P < 0.001, n = 3 versus normal cells, and ###*P* < 0.001 versus diabetic cells). One-way ANOVA was used to show statistically significant differences.

 Gelatin zymography was used for assessment of the modifications in MMP-2 and 9 activity in secretomes collected from the normal, diabetic and DFO treated diabetic ADSCs. The gelatinolytic activity of 72-kDa MMP-2 as well as 92-kDa MMP-9 of secretomes collected from normal ADSCs considerably elevated in comparison to diabetic ADSCs (*P* < 0.001). Results indicated significant enhancement of MMP-2 and 9 activity of ASC secretomes by DFO preconditioning in a concentration dependent way (*P* < 0.05 in the secretomes of 150 μM DFO preconditioned cells and *P* < 0.001 in the secretomes of 300 μM DFO preconditioned cell (Figure 5I and II)).

 Along with this result, Najafi et al have reported that preconditioning of BMSCs with Deferoxamine enhanced MMP-2 and -9 activity through a HIF-1-dependent manner.^37^

###  Condition medium from DFO treated ADSCs promoted HUVEC migration

**Figure 6:**
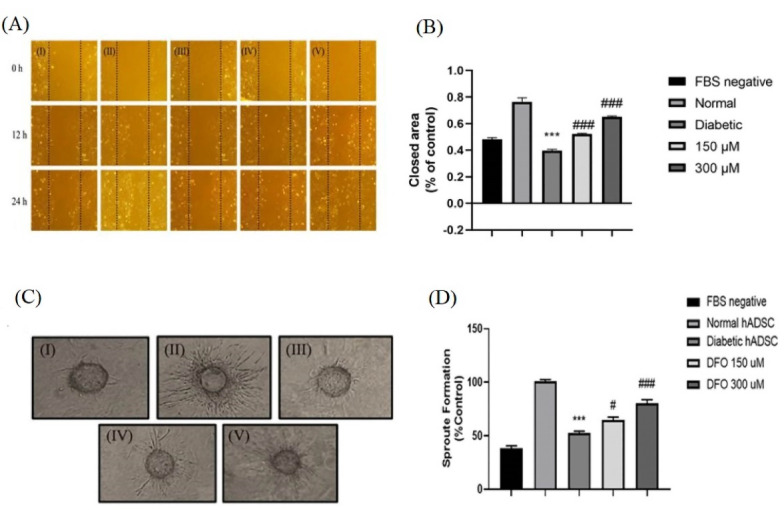
Evaluation of the angiogenic potency of the conditioned media extracted from the ADSCs. (A) Characteristic image of migrating the HUVECs cultured in various media. (I) medium with no FBS, (II) CM extracted from the normal ADSCs, (III) Diabetic ADSCs, (IV) 150 μM, (V) 300 μM DFO treated ADSCs by the inverted microscopy after 24 hours. (B) Evaluation of the regions without HUVECs using the NIH image software in 5 individual fields. (C) Inverted microscopy after 48 h was used to monitor the sprout formation of the HUVECs in (I) the medium without serum, (II) CM extracted from the normal ADSCs, (III) diabetic ADSCs, (IV) 150 (V) 300 μM DFO-treated diabetic ADSCs. (D) Changes in the sprout formation of the HUVECs were defined by NIH images and data were expressed as the mean ± SEM (***P < 0.001, **P < 0.01, n = 3 versus the normal cells and ### P < 0.001, ## *P* < 0.01 vs. the diabetic cells). One-way ANOVA was employed to measure statistical significance.

 Using an in vitro scratch assay, the paracrine effects of ADSCs were examined using the ADSC-conditioned media on the migration of endothelial cells. Results indicated that CM extracted from the normal (*P* < 0.001), 150 μL (P < 0.001) as well as 300 μL (*P* < 0.001) DFO treated diabetic ADSCs considerably elevated the HUVECs’ migration as compared to the CM extracted from nontreated diabetic ADSCs (Figure 6A). This was consistent with Mehrabani et al findings and supported the contention that CM derived from DFO preconditioned ADSCs manifest increased proangiogenic.^20^ Similarly, Ding et al reported that exosomes originated from BMSCs preconditioned by deferoxamine showed enhanced proangiogenic properties in the scratch wound healing assay.^51^

###  DFO elevated the tube formation in a 3D model of angiogenesis

 The CM collected from cultured normal ADSCs exhibited superior angiogenic property in induction of sprout formation compared to CM extracted from diabetic ADSCs (*P* < 0.001). Moreover, our results revealed that diabetic ADSCs treated with 150 μL (*P* < 0.05) as well as 300 μL (*P* < 0.001) DFO exhibited greater angiogenic capacity compare to nontreated diabetic ADSCs (Figure 6C).

 Tube formation assay showed that a greater number of cord-like structures were formed on Matrigel in DFO treated group in comparison to the nontreated diabetic group. Thus, DFO may cause diabetic ADSCs to secrete CM to show augmented proangiogenic potential in cell-free therapeutic applications.

## Conclusion

 In conclusion, preconditioning of diabetic ADSCs with Deferoxamine significantly enhances HIF-1α expression and the respective down-stream angiogenic genes such as FGF-2, SDF-1α, MMP-2 and -9 activity and VEGF, suggesting that autologous cell therapy efficiency of these cells would be elevated compared to normal ADSCs. Further investigation is needed to standardize cell extraction protocols and overcome the obstacles in diabetic-related stem cell yield and proliferation capacity.

## Acknowledgements

 Iran University of Medical Sciences supported the research and the authors kindly thank the contributions of Mrs. Neda Tekiyeh Maroof for her valuable technical assistance.

## Competing Interests

 Hereby, it is declared that we do not have any conflict of interests on the publication of the paper.

## Ethical Approval

 Not applicable. Each procedure and material used in the research was verified by the ethical committee of Iran University of Medical Science. The subjects also signed the informed consents.

## Funding

 This work was supported by a grant number 28638 from Iran University of Medical Sciences.
